# Decoding the perceived value of Wushu short videos and its influencing factors: an interpretable machine learning analysis based on TikTok videos

**DOI:** 10.3389/fspor.2026.1688807

**Published:** 2026-03-05

**Authors:** Yang Chen, Han Wu, Shang Xiang

**Affiliations:** 1 Department of Physical Education, Xiangtan University, Xiangtan, Hunan, China; 2 School of Public Administration, Xiangtan University, Xiangtan, Hunan, China

**Keywords:** 5W communication model, machine learning, perceived value, TikTok, Wushu short videos

## Abstract

**Introduction:**

Short video platforms have become a key medium for the dissemination of Chinese Wushu culture. However, audience perceptions of Wushu short videos remain underexplored. This study aims to explore how audiences perceive value in Wushu short videos.

**Methods:**

Drawing on perceived value theory and Lasswell's 5W model, this study examines how communicator competence, narrative elements, multimodal features, and audience cultural background collectively shape the perceived value of Wushu short videos. We collected 412 Wushu short videos and 236,627 user comments from TikTok, and analyzed the data using machine learning methods.

**Results:**

First, through topic clustering and sentiment analysis, we identified three perceived value dimensions: knowledge expansion value, physical and health value, and emotional experience value. These dimensions were unevenly recognized: emotional experience value received the most positive feedback, while knowledge expansion and health values were less acknowledged. Second, LightGBM regression and SHAP interpretability analysis showed that audience cultural background had the strongest impact on value perception, with cultural diversity as the most critical predictor. Communicator competence was the second most influential factor, in which social capital exhibited a negative correlation. Among narrative elements, traditional Chinese Wushu artist imagery enhanced perceived value, and female or mixed-gender presentations performed better than male-only presentations. Multimodal features showed limited influence.

**Discussion:**

These findings enrich the theoretical research on Wushu cultural communication. They also provide practical guidance for content creators to optimize Wushu short video dissemination strategies, including cultural image presentation, gender diversification, publishing strategies, and community building.

## 1 Introduction

In recent years, digital media has fundamentally reshaped cultural consumption behaviors among global audiences (1, 2). Digital media has become integral to daily cultural life, offering platforms where diverse content can be ubiquitously accessed and created (3). Short video platforms exemplified by TikTok, now serve over 160 countries with more than one billion monthly active users (4). These platforms disseminate diverse content—from music and dance to fitness—to global audiences (5–8), thereby establishing a digital cultural communication ecosystem (9). Wushu, the Chinese martial arts, as a crucial component of Chinese traditional culture, integrates philosophical thought, physical techniques, and cultural values (10). Representing the convergence of traditional wushu culture with contemporary media, Wushu short videos primarily feature traditional forms demonstrations, technical instruction, and contemporary performances (11). Beyond showcasing aesthetic value and stimulating audience participation (11, 12), these videos transmit Chinese cultural connotations (13–15). With platform globalization, Wushu short videos have attracted substantial global viewership through their distinctive cultural appeal (11). Consequently, they have emerged as a significant cultural communication phenomenon in the digital era.

Despite their growing influence, Wushu short videos have received limited scholarly attention. Existing Wushu cultural research primarily examines traditional media content—films and television dramas featuring classic figures like *Huang Feihong*, *Ip Man*, and *Bruce Lee*. These researches explore their macro-level functions in promoting Wushu tourism (16, 17) and facilitating international cultural exchange (18, 19). The emerging format of Wushu short videos has received insufficient scholarly attention. Meanwhile, sports short video research has examined audience interaction behaviors with various content types, including physical exercise videos (20), sports science courses (21), and athlete videos (22). These studies demonstrate that short videos serve as crucial media for sports cultural communication (23), with audience comprehension and acceptance proving pivotal for communication effectiveness (24). However, this body of research focuses predominantly on mainstream sports, paying minimal attention to Wushu short videos. Consequently, Wushu cultural studies and sports video research remain disconnected, leaving audience comprehension and acceptance largely underexplored. This gap limits current understanding of how effectively Wushu short videos communicate cultural content.

Audience acceptance of Wushu short videos fundamentally depends on how they perceive and evaluate content value. Rooted in consumer behavior research (25, 26.), perceived value is now widely used to explain why audiences select, consume, and share digital content (27, 28). Research indicates that audience perceived value of digital content encompasses multiple dimensions—functional, emotional, and social values (29–31). Collectively, these values drive content interaction and dissemination behaviors (32). Given the complexity of factors influencing perceived value, Lasswell's 5W communication model provides an appropriate analytical framework (33). Lasswell conceptualized communication through five key elements—who communicates, what is communicated, through which channel, to whom, and with what effect (33)—and subsequent scholarship has operationalized this framework for contemporary media contexts (34). Building on this theoretical integration, this study positions audience perceived value as the core measurement of communication effects and examines how several communication elements shape value perceptions of Wushu short videos, including communicator competence (35, 36), narrative elements (37–39), multimodal features (40–42), and audience cultural background (43, 44). This study is guided by the following two research questions:

RQ1: What value perceptions do audiences exhibit toward Wushu short videos?
RQ2: How do different communication elements influence audience perceived value of Wushu short videos?

To address these research questions, this study adopts TikTok as the analytical platform and employs machine learning methodologies for analysis. The dataset comprises 412 Wushu short videos and their associated 236,627 user comments collected from the TikTok platform. The analysis consists of two primary components. First, BERT-based pre-trained models are utilized for topic clustering and sentiment analysis (45, 46). Topic clustering is used to automatically extract the underlying dimensional structure of audience value perceptions from large-scale comment data, while sentiment analysis is employed to quantify the emotional attitudes associated with each identified value dimension. Second, a LightGBM regression model is implemented to capture nonlinear interactions among multiple variables (47). SHAP interpretability techniques are integrated into the model to address the “black box” problem (48). This approach provides detailed insights into the specific mechanisms through which each factor operates and quantifies their marginal contributions.

This study makes several theoretical and practical contributions. First, we identify and delineate the multidimensional structure of audience perceived value in Wushu short videos, thereby advancing the theoretical understanding of value perception in sports culture content. Second, we uncover the complex ways in which communicator competence, narrative elements, multimodal features, and audience cultural background jointly shape perceived value, providing a robust empirical foundation for the digital dissemination of Wushu and related sports cultures. Third, by integrating BERT-based semantic analysis, LightGBM modeling, and SHAP interpretability, this study introduces machine learning to communication effects research and offers a replicable analytical framework for future inquiries. From a practical standpoint, the findings yield actionable content optimization strategies for Wushu short video creators—such as maintaining regular posting schedules, emphasizing cultural authenticity, and diversifying gender representation—that enhance audience perceived value and facilitate the effective dissemination of Wushu culture on digital platforms.

## 2 Theoretical background and literature review

Lasswell's 5W model (33) provides a framework for dissecting how communicator, message, channel, audience, and effect jointly shape communication outcomes. It encompasses roles such as sender, message, channel, receiver, and effect to systematize the communication process. This model dissects the communication process through five interrogative dimensions—who says what, through which channel, to whom, and with what effect—providing a comprehensive analytical framework for the construction, dissemination, and reception of information in social contexts. Originally applied to mass communication, journalism, and propaganda, the framework is now increasingly used to analyze digital media and user-generated content (34). However, in recent years, its application has extended to digital media and user-generated content analysis. For example, one study applied the 5W model to examine the dissemination of health information on social platforms. From this, the researchers developed an eight-element communication model for online health rumors (49). Furthermore, researchers have applied the 5W model to the 24-h livestream domain to explore the communication dynamics and effects of cabin hospital construction livestreams during the COVID-19 pandemic (50). These advances highlight the model's enduring relevance and adaptability in analyzing contemporary digital communication dynamics (51). Lasswell's 5W model is particularly suitable as this study's theoretical foundation, given its comprehensive scope and flexibility in capturing interactive elements. It will be used to investigate the influencing factors and effects of audience perceived value of Wushu short videos on TikTok. Accordingly, the model for this study comprises five components: Audience perceived value, Communicator competence, Narrative elements, Multimodal features, and Audience cultural background.

### 2.1 What effect: audience perceived value

In the 5W communication model, communication effect is the core dimension for evaluating the effectiveness of communication activities. In the digital media environment, user interaction metrics such as view counts and likes are easily measurable. However, they fail to deeply reflect the audience's genuine understanding and acceptance of content (52). Consequently, communication effect research has gradually shifted from focusing on the direct impact of information to emphasizing audience subjective experience and evaluation. In this research transition, perceived value has emerged as an important perspective for understanding audience subjective experience. As a core concept in consumer behavior research (25, 26.), perceived value refers to the utility judgment formed by audiences based on their overall assessment of content (53). Central to this concept is the multidimensionality of value perception: audiences assess content across functional, emotional, and social dimensions (54, 55). Perceived value is also inherently subjective and context-dependent, such that the same content can elicit divergent value perceptions among different audiences (53).

In digital media research, perceived value has been demonstrated as a key variable in explaining user behavior. Studies show that perceived value directly affects users' intention to acquire, continue using, and recommend digital content (32, 56). Focusing specifically on video as a content format, users' value perception tends to be multidimensional. It encompasses the utilitarian value of information acquisition, the hedonic value of obtaining pleasure, and the social value of social sharing (31, 57). Existing research in the sports video domain has identified several value dimensions, including information quality, entertainment value, and group identity (20, 58, 59). However, the unique value structure of Wushu short videos as a specific genre remains systematically unexplored. Wushu short videos integrate multiple attributes, including sports skill demonstration, artistic aesthetic expression, and cultural connotation transmission. This unique combination potentially creates distinct dimensions of audience value perception that differ from those of general sports videos. Therefore, exploring the dimensions of audience perceived value for Wushu short videos and measuring their emotional attitude will contribute to a deeper understanding of the communication effects of Wushu short videos.

### 2.2 Who: communicator competence

Communicators determine both the content and the channel through which it is delivered. In digital media environments, their competence hinges on two core elements: content supply and social capital. Content supply serves as a key indicator of a communicator's content production capability (35). Users with extensive content publication records may more readily achieve widespread information dissemination on social media, as increased information sharing enhances users' hedonic experience and thereby cultivates their continuous participation behavior (35). However, alternative perspectives suggest that communicators who share substantial amounts of content may indicate insufficient creative resources. This can potentially weaken followers' perception of content novelty and reduce their engagement (60). Social capital centrally embodies communicators' social influence. Social capital, typically represented by follower count (61), has been demonstrated as an important factor influencing information circulation efficiency (36). Generally, a communicator's follower base is positively correlated with the interaction effects of their disseminated content (62). Nevertheless, eye-tracking experiment has revealed a complex inverted U-shaped relationship between communicators' follower count and their content interaction (63). These findings indicate that the influence of communicator characteristics in digital media communication remains contentious. Therefore, we attempt to investigate their potential impact on audience perceived value of Wushu short videos.

### 2.3 Says what: narrative elements

Communication content serves as the core carrier connecting communicators and audiences Its characteristics are what ultimately determine audience behavioral intentions. Narrative theory provides a crucial perspective for understanding video content characteristics. Narrative represents a universal human mode of expression that permeates all levels of communication activities (64). From this perspective, video communication essentially involves conveying information to audiences through storytelling. Research shows that narrative enhances video interactivity and vividness (65). Simultaneously, it imbues information with logical and emotional connections (66). Together, these effects strengthen the appeal of the content. In social media environments, exceptional video works typically attract audience attention through the organic combination of different narrative elements to facilitate sustained viewing behaviors (67). Building upon this foundation, we characterize the content features of Wushu short videos from a narrative perspective.

Characters, scenes, and themes constitute commonly recognized important narrative elements (68). Characters, representing persona images, link the logical relationships throughout the narrative process and hold significant meaning for audience emotional identification (69). In video communication, character can be depicted through gender and cultural image. Gender characteristics exert substantial influence on video communication effectiveness. For instance, evident correlations exist between video creators' gender differences and viewers' evaluative responses (70, 71). This suggests that the gender characteristics of artists in Wushu videos may differentially impact their communication efficacy. Meanwhile, the presentation of cultural image represents another dimension for characterizing Wushu video content features. In cultural communication contexts, different ethnic cultural images can evoke varying emotional responses from audiences, potentially triggering cultural misreading or exoticism phenomena (72, 73). This has been examined in racial representation of animated characters (74) and American influencer phenomena on Chinese platforms (75).

Scenes provide the spatiotemporal context that supports narrative development. They furnish the physical setting for stories while embedding content within specific cultural frames and emotional tones (68), thereby directly shaping narrative effectiveness. Narrative scenes can be defined along two dimensions—scene type and scene complexity. Scene type refers to the visual subject of the video narrative. Varied scene configurations afford audiences different forms of spatial imagination, which in turn yield differential communication potential (76). For example, research indicates that natural landscapes, compared to urban scenes dominated by architecture, more effectively stimulate preferences and evoke positive emotions (77). Scene complexity measures the quantity of visual elements within videos. Studies reveal that more complex scenes can enhance audience attention and stimulate more vivid psychological imagery (78, 79). Building on these, scenic elements in Wushu short videos may shape audience value perceptions: scene type, such as a natural landscape versus a formal Wushu venue, and scene complexity, which is often reflected in the scale of the Wushu performance.

Themes represent the core content and meaning expression of narratives. Thematic analysis can be developed along two dimensions: theme type and theme depth. Theme type manifests as content subject orientation, directly influencing audience acceptance of video content (80). Taking the Bilibili platform as an example, research found that different theme types such as content narratives and soft news demonstrate marked differences in attracting and maintaining audience attention (81). Wushu short videos similarly present a rich variety of theme types, including event documentation, character interviews, and detailed demonstrations (82). This variety suggests that audiences may have differential value perceptions for different types of Wushu short videos. Simultaneously, even within the same video type, differences persist in theme depth of thematic interpretation. Some research argues that in-depth thematic expression often demonstrates higher credibility and authenticity (83). This, in turn, helps construct systematic cognitive frameworks for viewers (84). However, opposing views contend that overly detailed content requires audiences to invest more cognitive resources in information processing, potentially creating comprehension challenges (85). Focusing on Wushu short videos, the wushu culture they communicate often encompasses multi-layered representations from material to institutional to spiritual dimensions (86). These depth differences may influence audience perceptual judgments regarding Wushu video value (87).

### 2.4 In which channel: multimodal features

Channels serve as the medium carriers through which content is delivered to audiences. In the digital media environment, videos are encoded and transmitted through multimodal channels, incorporating multiple information modalities such as text, visual, and auditory elements (88). Therefore, these modalities are treated in this study as the fundamental features of the channel, representing the symbolic forms through which the message is conveyed. Multimodal communication possesses distinct functional advantages. It enables a more efficient organization of complex information content (89) and also accommodates diverse audience perceptual preferences to stimulate more active interactive behaviors (90). From this perspective, Wushu short videos, as typical multimodal information combinations, create complete cultural communication experiences through visual action demonstrations, background music rendering, and textual explanatory supplements. The information presentation across different modalities may influence Audience Perceived Value.

Text serves as the fundamental information carrier in videos, providing semantic interpretation for visual content. Textual information in videos primarily exists in thematic tags and subtitles. Tags, functioning as keyword identifiers for video content, assist users in searching, following, and sharing information (91). The optimized configuration of tags exerts significant influence on video communication effectiveness (92). For instance, empirical analysis of YouTube videos revealed sensitivity between tag characteristics and video social traffic (93). Subtitles provide auxiliary explanatory descriptions of video content. They hold crucial significance for audiences with different linguistic and cultural backgrounds in understanding the content. Research demonstrates that video subtitles directly affect English learners' comprehension levels of video content (94) and produce significant impacts on foreign language learners across three dimensions of vocabulary knowledge: form, meaning, and usage (95). Therefore, both tags number and subtitle type may influence audience understanding of Wushu short videos content, subsequently affecting their value perceptions.

Visual elements constitute the most intuitive and prominent information carriers in videos. They are crucial in determining the attractiveness of video imagery (96). Visual characteristics primarily encompass brightness and richness. Brightness, as a pixel-level basic color characteristic, describes the color luminosity of video images and relates to audience visual experience (97). High-brightness visual content demonstrates positive correlation with visual working memory performance (98) and better stimulates audience content interaction behaviors (99). Visual richness describes visual characteristics from the perspective of color complexity. In essence, it reflects the information density within unit frames (97). Visual richness exhibits complex mechanisms regarding audience engagement behaviors in videos. Some research indicates that video visual richness maintains an inverted U-shaped relationship with user engagement behaviors (100). Another study focusing on Sina Weibo found positive correlations between richness and audience emotional and behavioral engagement (101). These evidences suggest that visual brightness and richness, as important visual characteristics of Wushu short videos. Therefore, they may influence audience perceived value.

Auditory characteristics embedded within videos include tempo and loudness, which can directly influence audience perceptual experiences of content (89). Audio tempo reflects the patterns of sound variation across temporal dimensions and relates closely to audience emotional experiences and content engagement levels (89, 102). For example, in tourism short video communication, fast-tempo background music can stimulate audiences' instantaneous attention capture. This subsequently influences their travel intentions (103). Audio loudness represents the direct manifestation of video sound energy, describing the intensity levels of audio signals. Loudness configuration can assist in attracting audience attention (104). Research on hotel short videos confirmed that appropriate loudness levels can effectively stimulate audience active participation behaviors (41). Therefore, we attempt to investigate the potential influences of audio tempo and loudness on audience perceived value of Wushu short videos.

### 2.5 To whom: audience cultural background

Audiences are the agents who receive communication content and actively construct its meaning. When Wushu videos circulate on global platforms like TikTok, they give rise to inherently cross-cultural communication scenarios, wherein viewers from diverse cultural backgrounds interpret and engage with content through their own cultural cognitive frameworks (105). Understanding how audience cultural characteristics shape their perceived value of Wushu short videos is thus of critical importance.

Audience cultural background can be measured through cultural difference and cultural diversity. Cultural difference refers to the degree of disparity between the culture to which audiences belong and the culture embedded within communication content (106). Cultural difference affects people's communication patterns and information interpretation modes (107), subsequently influencing audience understanding and acceptance of digital content. This has been substantiated in studies of K-pop music transmission on YouTube (108) and hotel customer online reviews (109). Cultural diversity reflects the richness of cultural backgrounds within audience groups. In cross-cultural communication contexts, the impact of cultural diversity on communication effectiveness remains contentious. Some research suggests that Cultural Diversity may increase communication complexity, conflict, confusion, and ambiguity (44). This can impede effective information transmission (110). However, other studies hold contrary perspectives. Analysis of social networks among American business school students found that students from regions with higher Cultural Diversity more readily connect different groups and establish social connections (111). This indicates that audiences with multicultural backgrounds may expand content transmission scope.

**Figure 1:**
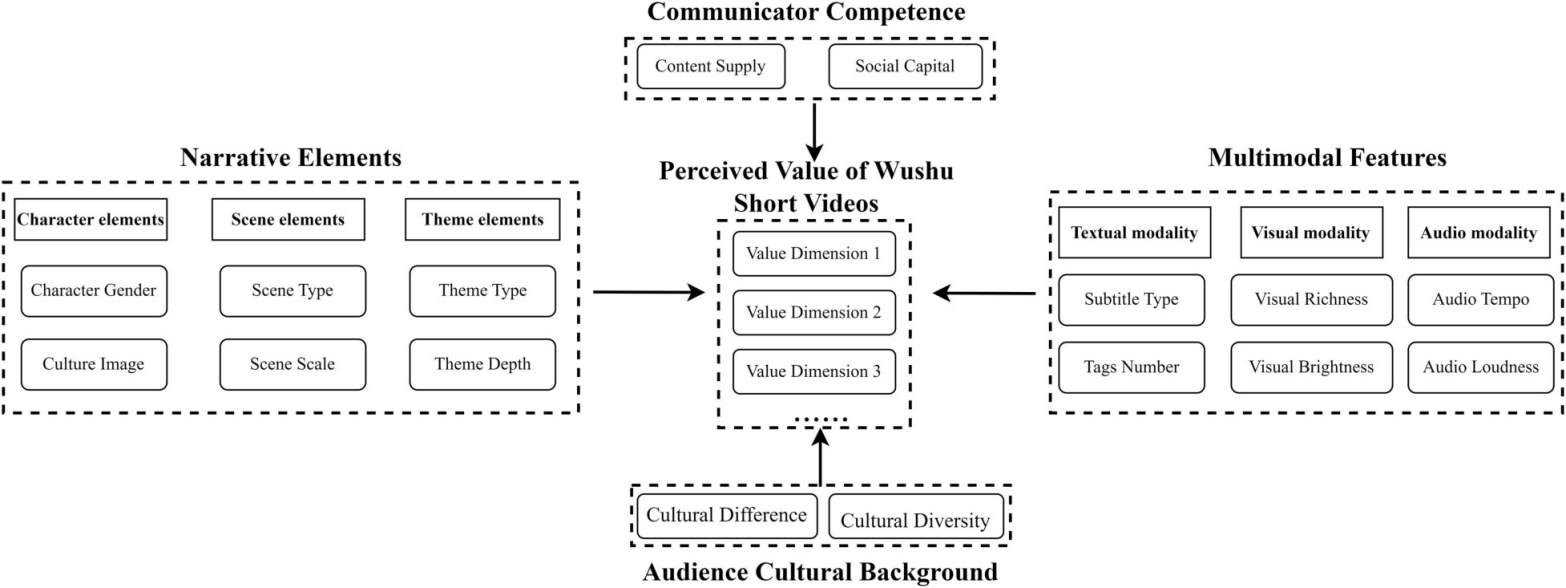
Model of wushu short video value perception determinants.

In summary, the aforementioned communication elements—Communicator competence, narrative elements, multimodal features, and audience cultural background—all potentially exert varying degrees of influence on audience perceived value of Wushu short videos. Based on this understanding, we propose an analytical model (Figure 1) and raise the following research questions:

RQ1: What value perceptions do audiences exhibit toward Wushu short videos?
RQ2: How do different communication elements influence audience perceived value of Wushu short videos?

## 3 Methodology

### 3.1 Data collection and sample

Our study focuses on the global dissemination of Wushu short videos and the cross-cultural audience perceived value associated with such content. Accordingly, the data were collected from TikTok (https://www.tiktok.com), which represents a highly typical global short-video platform. TikTok has established a substantial international user base, covering over 160 countries and regions with more than one billion monthly active users (4). Its algorithmic recommendation system and multilingual support further facilitate cross-cultural content circulation, enabling users from diverse cultural and linguistic backgrounds to engage with the same video content (112). While Wushu short videos are also disseminated on other platforms such as Kuaishou, Xiaohongshu, and Bilibili, these platforms primarily target domestic Chinese audiences. Therefore, focusing on TikTok allows this study to examine audience perceived value within a representative global short-video environment, which directly aligns with the research objectives.

We constructed the sample through a multi-step process. First, we defined a temporal boundary for data collection. Given the fast-evolving nature of TikTok's content ecosystem—where user interests and trending topics shift rapidly—the dissemination of Wushu short videos exhibits pronounced temporal patterns. Accordingly, we restricted our collection window to the six months immediately preceding the study. Second, we selected a set of globally recognized Wushu-related keywords as search queries, including “Kungfu,” “Wushu,” “Shaolin,” “TaiChi,” and “WingChun.” These terms were chosen for their international visibility and cultural resonance. Third, given that TikTok users can autonomously apply hashtags to their content, numerous videos bearing tags such as #kungfu were identified as irrelevant to Wushu content. To address this issue, two graduate students independently reviewed the retrieved videos. We followed established criteria for Chinese traditional sports culture (86). According to these criteria, valid videos were required to feature Wushu elements such as weaponry, notable practitioners, or specific martial arts disciplines. Discrepancies between the two reviewers were resolved by a third reviewer, ensuring the elimination of invalid content and maintaining sample quality. Furthermore, to ensure sufficient data for subsequent analysis, we filtered for videos with more than 100 comments. This step allowed us to capture content with high engagement levels and rich audience feedback. Through this rigorous screening process, we obtained 412 Wushu short videos. Using the Apify web scraping tool, we extracted video metadata and comment data, resulting in a comprehensive dataset of 236,627 comments.

### 3.2 Methods

#### 3.2.1 Topic clustering and sentiment analysis

To answer RQ1, we applied BERT-based topic clustering and sentiment analysis to 236,627 user comments, extracting latent value dimensions and their emotional valence. As direct expressions of audience cognition and emotion, user comments contain rich semantic and affective information (58, 113). Compared with simple interaction metrics such as likes and shares, comment texts provide more nuanced and multidimensional insights into how audiences perceive and evaluate video content. Therefore, machine learning–based text analysis is adopted to systematically extract latent value dimensions and emotional orientations from user comments.

With respect to method selection, traditional topic modeling approaches (e.g., LDA and LSA) primarily rely on word frequency statistics and are limited in capturing contextual semantics. Similarly, conventional sentiment analysis methods are often constrained by predefined sentiment lexicons, which restrict their effectiveness in multilingual environments. In contrast, BERT-based models leverage a bidirectional Transformer encoder architecture to fully exploit contextual information for deep semantic representation, effectively addressing the sparsity and ambiguity inherent in short text data (114). Moreover, multilingual BERT models enable cross-linguistic semantic alignment, making them particularly suitable for analyzing comments generated by culturally diverse audiences.

Specifically, this study adopted BERTopic as the topic clustering method. BERTopic integrates several machine learning steps, including sentence embedding, dimensionality reduction, density-based clustering, and class-based TF–IDF keyword extraction, to identify semantically coherent topics from large-scale text data (115). In this study, BERTopic was applied in an unsupervised manner to cluster user comments into latent thematic groups. Each resulting topic was subsequently interpreted as a distinct dimension of audience perceived value toward Wushu short videos.

To further capture audiences' emotional attitudes toward each value dimension, sentiment analysis was conducted using the “distilbert-base-multilingual-cased-sentiments-student” fine-tuned model (116). This model is specifically optimized for multilingual sentiment classification tasks and can effectively distinguish emotional polarity across different languages. Comments within each identified topic were classified into three sentiment categories: positive, neutral, and negative.

The analysis proceeded in two sequential phases. First, we applied BERTopic to extract latent dimensions of audience perceived value from the comment data. Second, we conducted sentiment analysis within each identified topic to quantify the emotional valence associated with each value dimension. The outputs from topic clustering and sentiment classification were then integrated to construct quantitative indicators of audience perceived value, which served as the target variables for the subsequent machine learning modeling.

#### 3.2.2 Interpretable machine learning

To address RQ2 regarding how communication elements influence audience perceived value of Wushu short videos, this study employs machine learning methods to construct a regression prediction model between communication elements and audience perceived value. The target variable in this model is the audience perceived value indicator derived from user comments in the previous analytical stage, while the independent variables consist of multiple communication elements, including communicator competence, narrative elements, multimodal features, and audience cultural background.

Unlike linear regression, which presumes predefined functional forms, machine learning autonomously uncovers hidden relational structures from large-scale data. This capability provides significant advantages in addressing complex and nonlinear relationships among high-dimensional data (117, 118). Accordingly, this study selects the LightGBM model for predictive modeling of audience perceived value. As an ensemble learning algorithm based on gradient boosting decision trees (47), LightGBM can automatically process different types of feature variables and enhance prediction accuracy by integrating multiple weak learners. Furthermore, its built-in regularization mechanism effectively prevents overfitting issues. These characteristics enable it to effectively handle the multidimensional communication element data involved in this study.

Although machine learning models can effectively identify complex relationships among communication elements, their internal decision-making processes are often difficult to intuitively understand, rendering them as “black boxes” (119). This limitation restricts their explanatory value in social science research. To address this constraint, SHAP (Shapley Additive Explanations) is incorporated into the analysis. Drawing upon Shapley value theory from cooperative game theory (48), SHAP quantifies the marginal contribution of each feature to the model's prediction. In this study, SHAP values are used to examine both the magnitude and direction of influence exerted by different communication elements on audience perceived value, thereby enabling a transparent and interpretable analysis of the underlying influence mechanisms. By combining LightGBM prediction results with SHAP explanations, this study preserves machine learning's ability to model complex relationships while simultaneously providing substantive insights into how different communication elements shape the audience's perceived value of Wushu short videos.

### 3.3 Variables

#### 3.3.1 Target variable

Audience perceived value represents the core target variable of this study, reflecting the degree of positive perceptions that audiences hold toward Wushu short videos across different value dimensions. Based on the aforementioned BERTopic topic clustering analysis, this research identified multiple dimensions of audience value perception. Through sentiment analysis, comments under each dimension were classified into three categories: positive, neutral, and negative. Building upon this foundation, it is necessary to construct quantitative indicators to measure audience perception levels across various value dimensions.

Solely using the number of positive comments is susceptible to the influence of the total comment volume. This leads to videos with larger comment bases having numerical advantages, while neglecting the relative intensity of value perception. Conversely, using only the proportion of positive comments captures relative positivity but ignores the breadth of value dissemination reflected in absolute quantities. To address these limitations, this study adopts the construction approach of the P-index (120) to overcome these limitations. By comprehensively considering both the absolute quantity and relative proportion of positive comments, we aim to authentically reflect audiences' actual perception levels of specific value dimensions in Wushu short videos. The calculation formula is as follows (see Equations 1 and 2):

$$P_{i}=\sqrt[{3}]{N_{i}\times R_{i}}$$ (1)

*P* represents the audience value perception level in the *i*-th dimension; $N_{i}$ denotes the number of positive comments in the *i*-th value dimension, and $R_{i}$ represents the proportion of positive comments relative to total comments in that dimension.

Given the varying importance of different value dimensions, we employ the entropy weight method to determine the weight $w_{i}$ for each dimension. The calculation is based on the distribution characteristics of positive comment proportions across all video samples in each value dimension. Accordingly, the comprehensive audience perceived value level for Wushu short videos is calculated as:

$$PV=\overset{m}{\underset{i=1}{\sum }}\;w_{i}\times P_{i}$$ (2)

#### 3.3.2 Feature variables

The feature variables are primarily categorized into four dimensions: Communicator competence, Audience cultural background, Narrative elements, and Multimodal features. Communicator competence features are extracted from the metadata of Wushu short videos. Audience cultural background features are identified using large language models to recognize the language of comments and calculate their distribution, which is then quantified by applying the. WALS database. Narrative elements are manually labeled, verified for encoding consistency, and subsequently input into LightGBM for direct transformation. Multimodal features are automatically computed using relevant Python libraries, including OpenCV and Librosa. The specific measurement methods are detailed in Table 1.

**Table 1:** Definitions, and measurement of the feature variables.

Influence Dimension	Feature variables	Measurement
Communicator Competence	Content Supply	Video posting volume of wushu short video publishers (continuous variable)
	Social Capital	The number of followers of wushu short video publishers (continuous variable)
Audience Cultural Background	Cultural Diversity	Use Spark4.0 Ultra large language model to identify comment text languages and count the number of language types (continuous variable)
	Cultural Difference	Based on audience comment language distribution and WALS database (121), calculate weighted linguistic distance of the audience (continuous variable)
Narrative Elements	Character Gender	Coded based on the gender characteristics of Wushu artists: Male 1, Female 2, Mixed 3 (categorical variable)
	Culture Image	Coded based on the cultural image of Wushu artists: Primarily Chinese 1, Primarily non-Chinese 2, Mixed 3 (categorical variable)
	Scene Type	Coded based on common Wushu scene categories: Natural scenery 1, Cultural landmarks 2, Professional venues 3, General scenes 4 (categorical variable)
	Scene Scale	Based on performer count in Wushu short videos: Solo 1, Duo 2, Group 3 (categorical variable)
	Theme Type	Coded based on Zhuang Wenjie et al.'s (82) video classification system: Event documentation 1, Character interviews 2, Detail demonstration 3, Voice-over narration 4, Situational recreation 5 (categorical variable)
	Theme Depth	Coded based on Lu Xing et al.'s (86) sports culture classification system: wushu material culture 1, wushu institutional culture 2, wushu spiritual culture 3 (categorical variable)
Multimodal Features	Visual Richness	Use OpenCV library to perform grayscale processing on short videos, measure the proportion of grayscale pixels in video frames, and calculate information entropy (89) (continuous variable)
	Visual Brightness	Use OpenCV library to perform grayscale processing on short videos, calculate the average value of pixels in grayscale images (89) (continuous variable)
	Audio Tempo	Use the “beat” module of Librosa library to derive the global tempo of each wushu short video (104) (continuous variable)
	Audio Loudness	Use Librosa library to extract audio signals from short videos, calculate the root mean square energy (RMSE) of audio signals (104)(continuous variable)
	Subtitle Type	Coded based on common subtitle settings in Wushu short videos, classified as: No subtitles 1, Chinese subtitles 2, non-Chinese language subtitles 3, Mixed subtitles 4 (categorical variable)
	Tags Number	Extract the number of # tags in Wushu short video titles (continuous variable)

## 4 Results

### 4.1 Audience perceived value of wushu short videos

#### 4.1.1 Dimensions of audience perceived value

**Figure 2:**
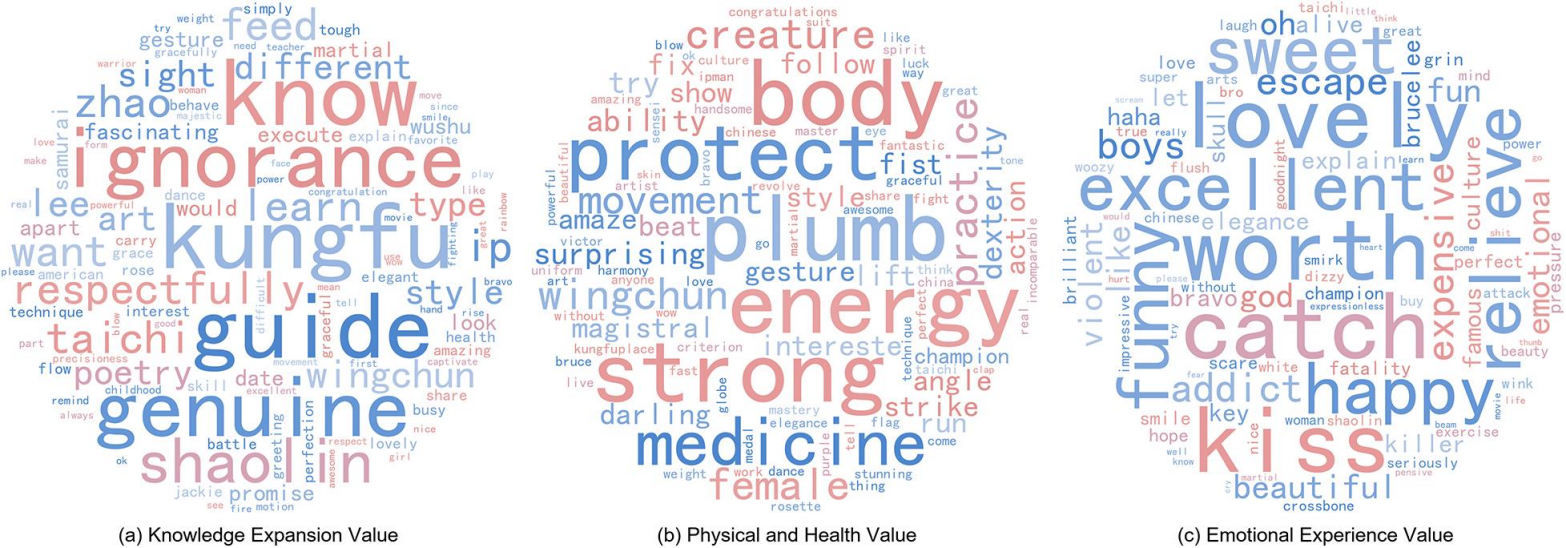
Determination of optimal number of topics.

To reveal the value perception dimensions of audiences towards Wushu short videos, this study conducted BERTopic through the following procedures. First, comments were transformed into 768-dimensional vectors using the “distilbert-base-multilingual-cased” model to capture deep semantic information. Subsequently, UMAP was employed to reduce dimensions to 10 while preserving key semantic features. Finally, K-means clustering was applied to automatically identify the latent dimensional structure of audience value perception. We determined the optimal cluster number by evaluating two complementary metrics for K values between 2 and 20 (Figure 2). The Silhouette Score was utilized to measure clustering consistency (122), whereas the Calinski-Harabasz Index was employed to evaluate inter-cluster separation and intra-cluster cohesion (123). Since both metrics indicate better clustering performance with higher values, they consistently indicated that K = 3 represented the optimal configuration, with the Silhouette Score reaching its maximum value of 0.7192 and the Calinski-Harabasz Index achieving 4,387.5.

**Figure 3:**
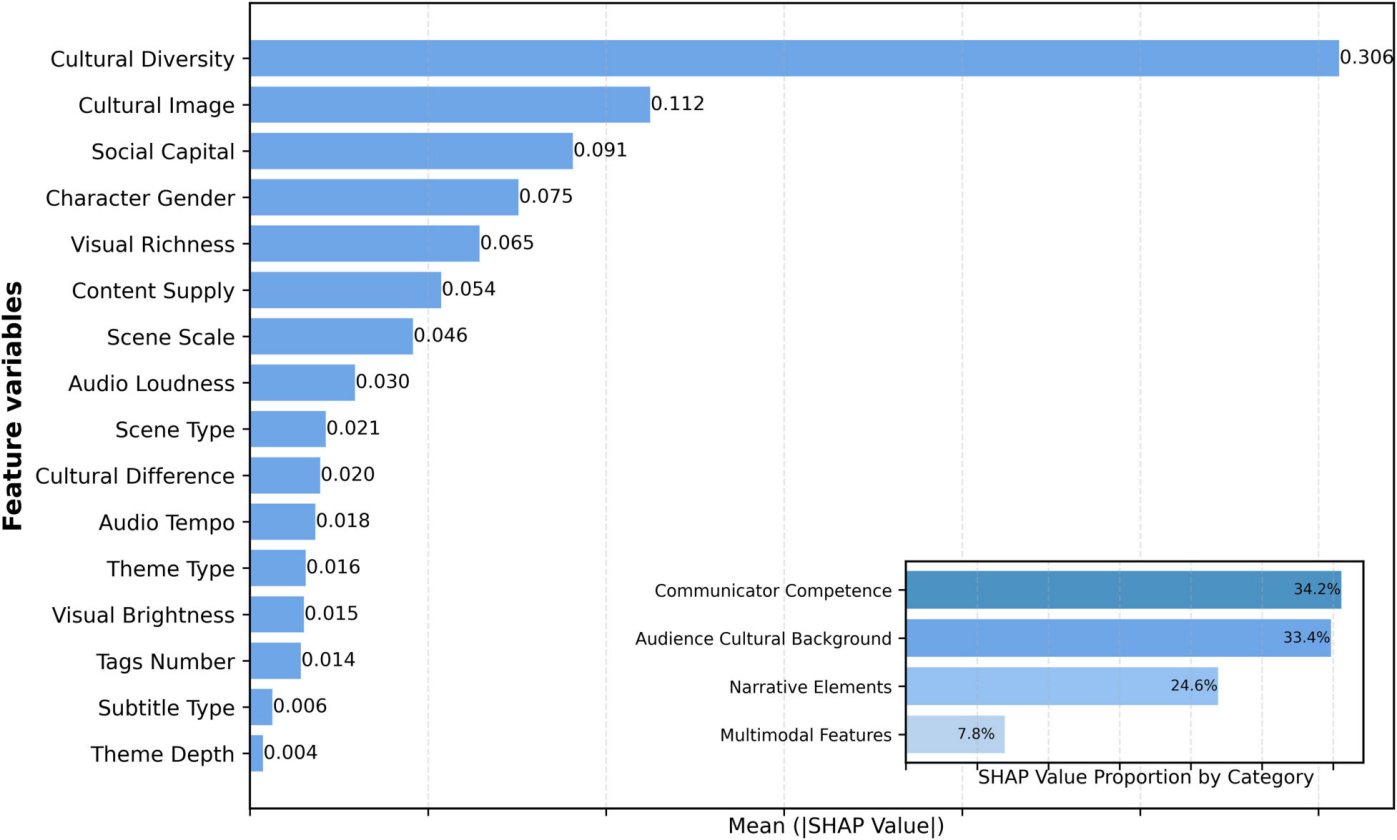
Dimensions of audience perceived value in wushu short videos.

Based on the keyword importance ranking, a word cloud visualization was employed for presentation (Figure 3). Analysis of Theme 1 reveals that specific Wushu disciplines constitute the primary focus of audience attention, with Wushu categories such as “shaolin” and “wingchun” attracting substantial audience interest. Concurrently, Wushu celebrities including “lee”, “jackie”, and “zhao” emerge as key figures through whom audiences understand Chinese Wushu culture. The high-frequency usage of terms such as “know”, “guide”, and “explain” indicates that audiences not only focus on the performative aspects of Wushu but also demonstrate a strong desire to acquire information regarding Wushu techniques and philosophical connotations. Their interest in mastering Wushu skills through learning “skill” and “technique” is evident. Therefore, Theme 1 is categorized as “Knowledge Expansion Value”. This theme suggests that audiences progressively construct systematic cognition of the Wushu cultural system through their attention to Wushu schools, figures, and techniques, thereby generating value recognition at the knowledge level. The frequent occurrence of knowledge-inquiry vocabulary in comments reflects that audiences have transitioned from mere visual appreciation to active exploration of Wushu culture.

In Theme 2, audiences focus on specific performance details of Wushu. Keywords such as “gesture”, “fist”, and “action” in the word cloud indicate heightened audience attention to Wushu movements, particularly manifested in technical observation. This also reflects the cultural influence of Wushu as a form of “bodily artistry”. Terms including “body”, “strong”, and “protect” reflect audience attention to the health value of Wushu, demonstrating their understanding of the positive health effects associated with Wushu practice. Terms such as “medicine” and “energy” further underscore this aspect. Moreover, the appearance of terms like “try”, “practice”, and “follow” indicates audiences' active willingness to participate in Wushu training. Consequently, Theme 2 is categorized as “Physical and Health Value”. This theme highlights audience recognition of the health benefits of Wushu and their tendency to transform cultural identification into practical engagement. Comments encompass both rational cognition of Wushu's health value and specific practice intentions. This transformation from cognition to practice suggests that Wushu culture has progressively taken root in audiences' daily lives.

Theme 3 demonstrates that positive emotional experiences constitute a significant motivation for audiences viewing Wushu short videos. Keywords such as “beautiful”, “lovely”, and “excellent” in the word cloud reflect audience recognition of character images and Wushu performance skills presented in short videos. Terms including “relieve”, “fun”, “smile”, and “happy” indicate that Wushu short videos not only possess entertainment value but also assist audiences in alleviating stress and enhancing emotional states. This generates evaluations such as “worth” and “addict”, indicating sustained attention tendencies toward Wushu short videos. Therefore, this theme is categorized as “Emotional Experience Value”. Compared to the previous two themes, the value perception embodied in this theme is relatively superficial, yet it demonstrates that audiences are attracted by the artistic expressiveness of Wushu and thereby obtain positive emotional experiences. This emotional identification based on artistic aesthetics becomes a crucial factor driving the widespread dissemination of Wushu short videos across different cultural contexts.

#### 4.1.2 Emotional attitudes toward each value dimension

**Figure 4:**
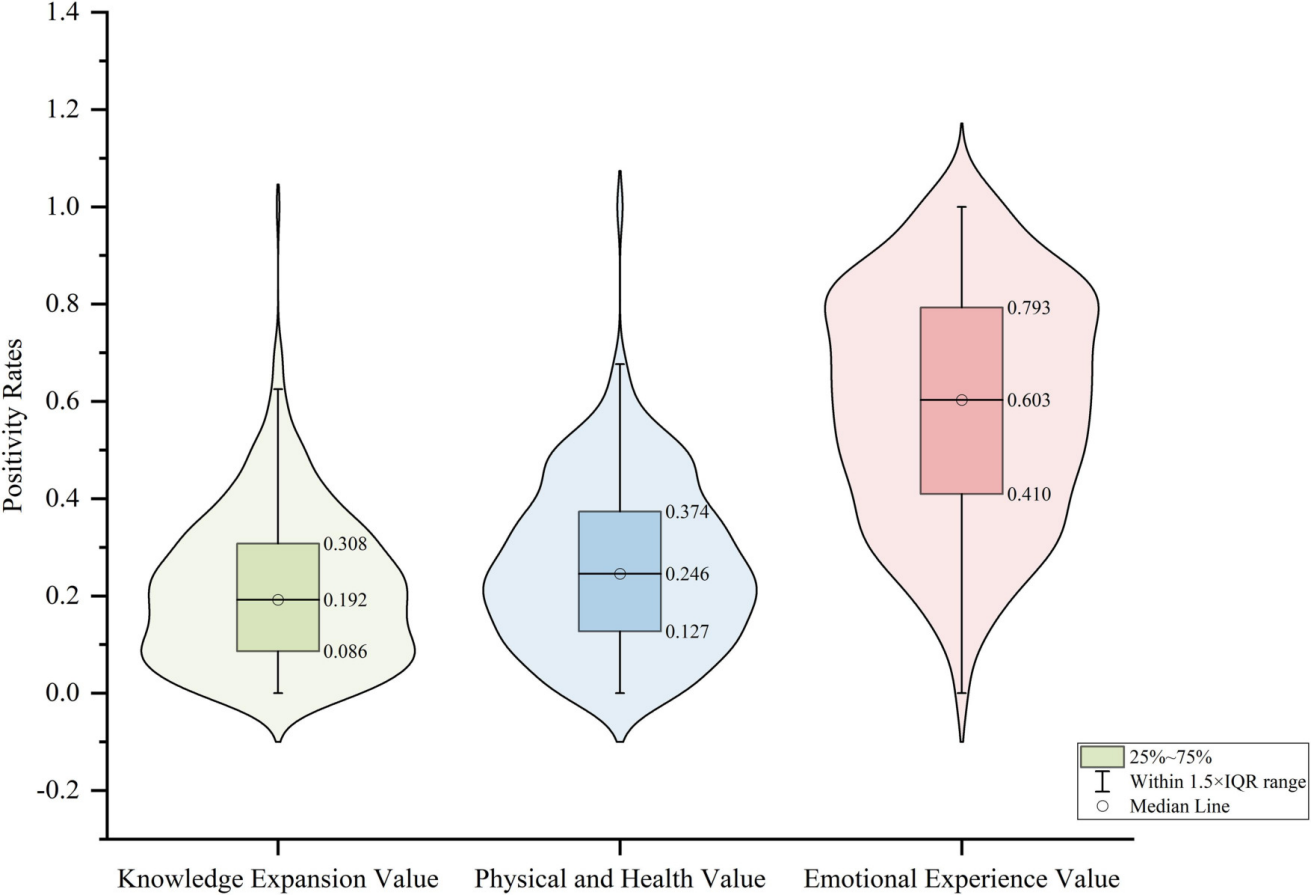
Positive sentiment ratio distribution across three value dimensions.

To further explore audience emotional attitudes toward different value dimensions of Wushu short videos, a multilingual fine-tuned model was employed to annotate sentiment labels for each comment. Based on this annotation, the positive sentiment ratios were calculated across all video samples for three dimensions: Knowledge Expansion Value, Physical and Health Value, and Emotional Experience Value. Violin plots (Figure 4) visualize the distribution of audience emotional attitudes across the three value dimensions.

Examining the overall distribution characteristics, Knowledge Expansion Value received the lowest positive sentiment ratio (median = 0.192), with most comments falling between 0 and 0.4. The Physical and Health Value dimension demonstrates intermediate performance, with a median of 0.246 and data distribution ranging from 0.1 to 0.6. The Emotional Experience Value dimension shows the most prominent performance, with a median reaching 0.603, data primarily distributed across the 0.2–1.0 interval, and substantial data concentration in high-value regions. Regarding data dispersion, the Knowledge Expansion Value dimension displays the most concentrated distribution, with a relatively small interquartile range (0.086–0.308), indicating that most Wushu videos demonstrate relatively consistent yet generally low effectiveness in eliciting positive emotions through knowledge transmission. The Physical and Health Value dimension exhibits moderate dispersion, with relatively uniform data distribution. Although the Emotional Experience Value dimension achieves the highest overall level, it also demonstrates the widest distribution range, with a larger interquartile range (0.410–0.793), suggesting significant variation in emotional elicitation performance across different videos, where certain videos achieve high positive sentiment ratios while others perform moderately.

This distribution pattern reflects certain disparities in audience perception across different value dimensions of Wushu short videos. Audience positive responses to Wushu short videos primarily manifest at the emotional level, where the artistic nature and visual appeal of Wushu more readily evoke positive emotional experiences among audiences. In contrast, audience positive attitudes toward Wushu knowledge acquisition and fitness value recognition remain relatively limited, indicating that Knowledge Expansion Value and Physical and Health Value retain considerable potential for enhancement in audience perception.

### 4.2 Factors influencing audience perceived value

#### 4.2.1 Importance of feature variables

To ensure optimal performance of the LightGBM model, Bayesian Optimization was employed for hyperparameter tuning. Compared to traditional methods such as grid search, Bayesian optimization constructs a probabilistic model of the objective function, enabling more efficient exploration of the parameter space for optimal configurations (124]. In this study, all samples were randomly divided into training and test sets following an 80:20 split ratio. Using the Optuna framework (125), we performed Bayesian optimization on the training set by minimizing the five-fold cross-validation root mean square error (RMSE) to identify the optimal hyperparameter configuration. The primary tuning parameters included: learning_rate (0.01–0.1) to control convergence speed; tree complexity parameters max_depth (3–6) and min_child_samples (10–50) to regulate fitting capacity; subsampling ratio feature_fraction (0.60–1.00) to introduce randomness; and L1/L2 regularization strength (0–3) to mitigate overfitting.

The empirical results demonstrate that the LightGBM model achieved a coefficient of determination (R^2^) of 0.547 on the test set, indicating that the model explains 54.7% of the variance in audience perceived value, thus exhibiting satisfactory fitting performance. The root mean square error (RMSE) of 0.384 suggests that the deviation between predicted and actual values remains within a reasonable range.

**Figure 5:**
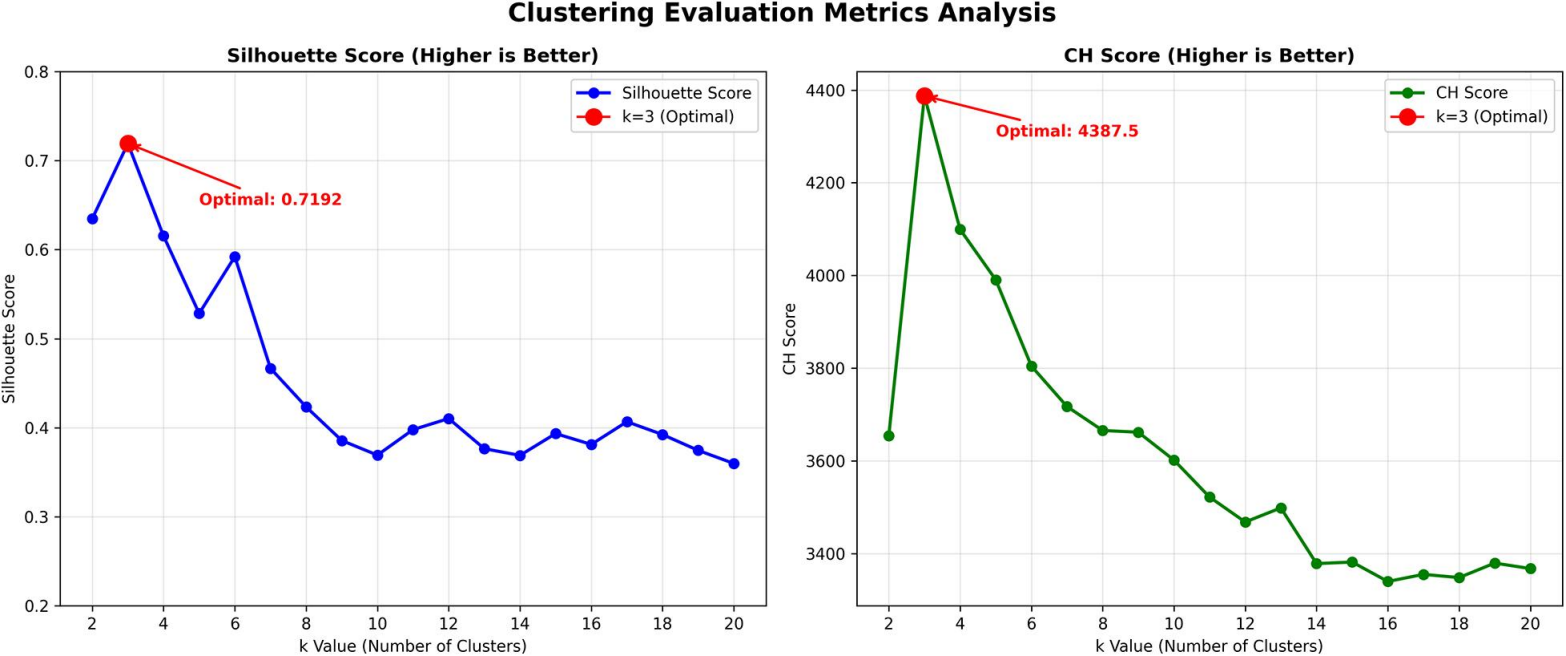
Feature importance ranking.

Based on the SHAP feature importance analysis results (Figure 5), distinct communication elements exhibit differential impacts on audience perceived value of Wushu short videos. Regarding the relative contributions of the four dimensions, Audience Cultural Background factors dominate with 34.2%, followed by Communicator Competence at 33.4%, Narrative Elements ranking third at 24.6%, whereas Multimodal Features demonstrate relatively limited influence at 7.8%. These findings indicate that when perceiving the value of Wushu short videos, audiences are primarily influenced by socio-cultural factors and communicator characteristics, while exhibiting relatively lower sensitivity to narrative elements and multimodal technical features.

The Audience Cultural Background dimension demonstrates the strongest explanatory power. Its most critical feature, Cultural Diversity, achieved a SHAP value of 0.306, substantially exceeding all others and emerging as the primary factor influencing audience perceived value. This suggests that the degree of cultural diversity among commenting audiences plays a substantially important role in their perception of Wushu short video value. In contrast, Cultural Difference exerts relatively minimal influence (0.020), indicating that the cultural distance between audiences and Wushu culture does not constitute a key constraining factor.

The Communicator Competence dimension contributes 33.4% of explanatory power, with Social Capital ranking third among all factors with a SHAP value of 0.091, while Content Supply demonstrates a SHAP value of 0.054. These results indicate that communicators' influence and content production capabilities represent important factors in shaping audience perceived value.

The Narrative Elements dimension contributes 24.6% of the total explanatory power. Within this dimension, Cultural Image exhibits the most prominent performance with a SHAP value of 0.112, ranking second in importance among all features. This suggests that the presentation of cultural identity in Wushu short videos significantly impacts audience perceived value. Furthermore, Character Gender demonstrates considerable influence (0.075), reflecting that audiences hold differentiated perceptions toward Wushu artists of different genders. Notably, the impact of Scene Scale (0.046) exceeds that of Theme Type (0.016) and Theme Depth (0.004), indicating that the scale of martial arts performance scenes influences audience value judgments more substantially than thematic content characteristics.

The overall impact of Multimodal Features remains relatively limited, contributing only 7.8% of explanatory power. Within this dimension, Visual Richness leads with a SHAP value of 0.065, demonstrating that visual richness exerts certain influence on audience perceived value. Audio Loudness (0.030) and Audio Tempo (0.018) play relatively minor roles, whereas Visual Brightness (0.015), Tags Number (0.014), and Subtitle Type (0.006) exhibit marginal effects.

#### 4.2.2 Effects of feature variables

**Figure 6:**
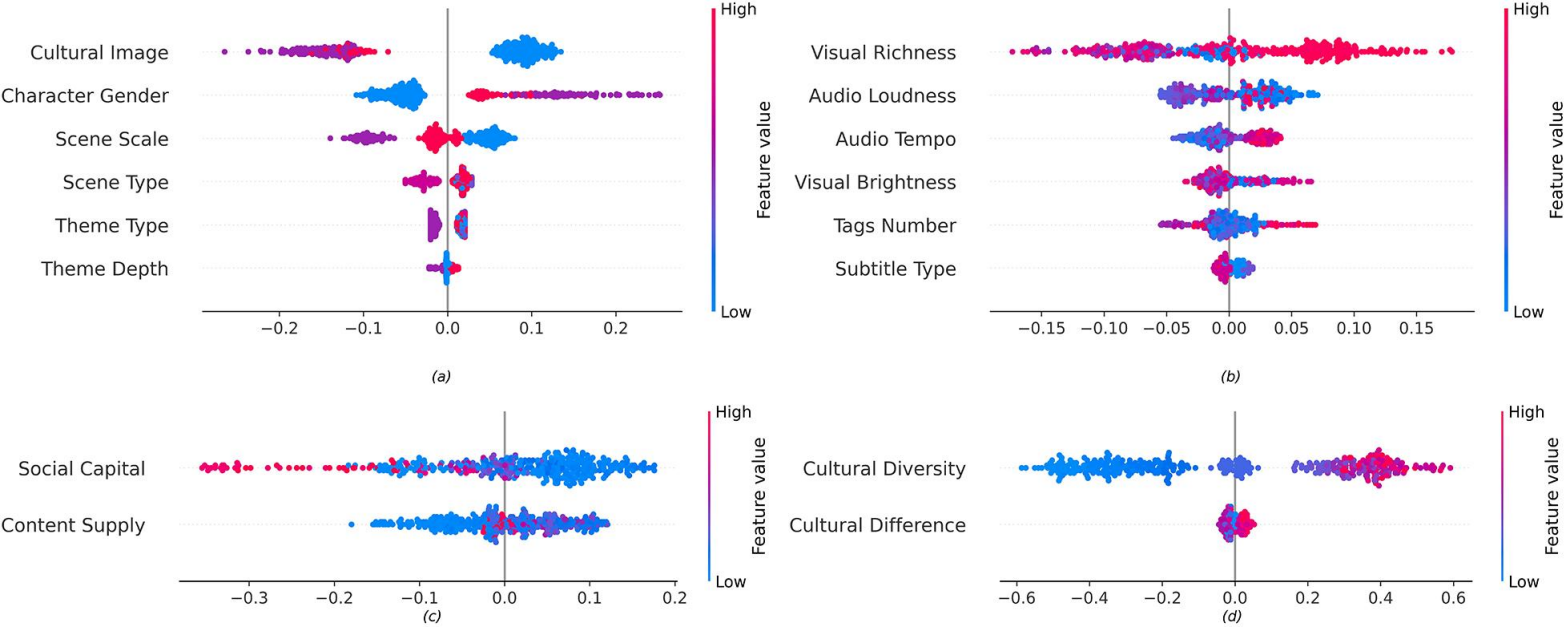
Summary of SHAP values for wushu short video value perception determinants. **(a)** Impact of narrative elements. **(b)** Impact of multimodal features. **(c)** Impact of communicator competence. **(d)** Impact of audience cultural background.

To further illustrate the directional effects of different feature variables on audience perceived value, SHAP value summary plots were generated (see Figure 6). As shown in Figure 6a, the SHAP value distribution for Cultural Image reveals that videos featuring predominantly Chinese cultural representations (value = 1) are concentrated in the positive region, whereas those featuring predominantly non-Chinese (value = 2) or mixed cultural representations (value = 3) are primarily distributed in the negative region. This indicates that Wushu short videos featuring Chinese Wushu artists are more conducive to enhancing audience perceived value. Regarding the Character Gender feature, male Wushu artists (value = 1) generate negative effects, while female Wushu artists (value = 2) and mixed-gender presentations (value = 3) produce positive effects. This suggests that, compared to traditional “male-dominated” Wushu displays, female participation or mixed-gender presentations are more effective in enhancing audience perceived value. The influence of Scene Scale is non-linear. While solo performances (value = 1) and group performances (value = 3) result in dispersed SHAP value distributions, two-person sparring (value = 2) generates more positive effects. This finding indicates that audiences have a stronger preference for two-person Wushu scenes. The SHAP values for Scene Type, Theme Type, and Theme Depth are concentrated near zero, with considerably smaller effect magnitudes than the aforementioned three variables, suggesting their relatively weak direct influence on audience perceived value.

As illustrated in Figure 6b, the Visual Richness feature shows most data points with high values (red/purple) on both positive and negative sides, though with a higher density in the positive region. This distribution indicates that high visual richness generally facilitates enhanced audience perceived value, albeit with certain instability. Visual Brightness displays a relatively uniform color distribution with mixed red and blue points, suggesting inconsistent directional effects on audience perceived value that may vary across videos. Concerning auditory features, Audio Loudness demonstrates a non-linear relationship, with medium volume levels producing negative effects while lower and higher volumes generate more positive effects. This U-shaped relationship indicates that extreme volumes may attract audience attention due to their distinctiveness. Audio Tempo presents a relatively clear positive correlation pattern, with SHAP values transitioning from negative to positive as tempo increases from slow to fast, suggesting that fast-paced background music enhances audience perceived value. Regarding textual features, the effect direction of Tags Number is unstable, with varying tag quantities potentially producing both positive and negative effects. Although its overall influence is relatively minor, the Subtitle Type feature reveals a clear pattern. Lower values (no subtitles = 1; Chinese subtitles = 2) correspond to higher SHAP values, while higher values (non-Chinese subtitles = 3; mixed subtitles = 4) correspond to lower ones. This pattern indicates that purely visual content or videos with Chinese subtitles are more effective in enhancing audience perceived value.

Figure 6c reveals a negative correlation between social capital and perceived value: creators with fewer followers tend to generate positive effects, whereas those with large follower bases are more likely to yield negative ones. This suggests that a high follower count is not a prerequisite for positive value perception of Wushu short videos. The effect of content supply is more nuanced. While low publication volumes (blue) are distributed evenly across both positive and negative SHAP regions, high publication volumes (red/purple) cluster predominantly in the positive region. This pattern indicates that more frequent content updates foster greater perceived value among audiences.

As illustrated in Figure 6d, the data for Cultural Diversity shows a clear pattern: low values are concentrated in the negative region, while high values are primarily distributed in the positive region. This distribution demonstrates a pronounced positive correlation, indicating that greater cultural background diversity among the audience corresponds to a more positive value perception of Wushu short videos. In contrast, the influence of Cultural Difference is relatively limited, with SHAP values concentrated near zero, suggesting that the distance between audiences and Chinese culture has virtually no direct impact on their value perception.

## 5 Discussion and conclusion

### 5.1 General discussion

Regarding RQ1, we identified a three-dimensional structure of audience perceived value for Wushu short videos: knowledge expansion value, physical health value, and emotional experience value. This finding aligns with prior work on the multidimensionality of perceived value (29, 55), while further revealing how Wushu short videos distinctively embed health preservation beliefs into modern media formats. Compared with general sports videos, our study discovered both similarities and differences. The knowledge expansion value emphasizes the extent to which audiences acquire Wushu-related knowledge from short video content. Meanwhile, the emotional experience value focuses on the emotional utility gained from watching Wushu short videos. These dimensions essentially correspond to the information value (58) and entertainment value (20) identified in previous research. Although health utility is a fundamental attribute of sports content, this dimension has not been independently extracted in previous sports video studies. Our findings indicate that audiences have explicit cognition of Wushu's fitness value, with health-related vocabulary such as “body,” “strong,” “protect,” and “medicine” suggesting that Wushu is perceived as a physical practice with health preservation functions. This perception may stem from the continuation of traditional health preservation concepts such as “yin-yang harmony” and “unity of heaven and human” in Wushu culture within digital media (87).

Notably, the three value dimensions exhibit significant differences in positive emotional expression. The emotional experience value demonstrates the highest proportion of positive emotions, far exceeding physical health value and knowledge expansion value. This emotional pattern reveals an imbalance in audience perceived value of Wushu short videos. The dominance of emotional value likely stems from the format of Wushu short videos: their brevity and visual intensity favor immediate affective response over deep cognitive elaboration (3). The visual aesthetics of Wushu movements can effectively trigger audience emotional resonance within a short time, thereby satisfying short video users' demand for quick entertainment during fragmented time (54). In contrast, although audiences demonstrate high interest in learning Wushu knowledge and practical participation, the limited content depth of short videos constrains the transmission of related complex Wushu information. This may account for the lower emotional polarity in these two dimensions.

Regarding RQ2, several phenomena warrant in-depth analysis when exploring factors influencing audience perceived value. First, cultural background factors occupy a dominant position in influencing audience perceived value. Cultural diversity emerges as the most important predictor among all factors, while the influence of cultural difference remains relatively weak. This finding contradicts traditional cognition, where cultural differences are typically viewed as the primary barrier to understanding and accepting different cultural content (105, 108). This contrast may arise because comment sections with diverse audiences construct an inclusive cross-cultural discourse space. This inclusive atmosphere reduces the psychological distance among different audiences and facilitates the generation and dissemination of positive emotions, thereby enhancing audiences' overall value perception. Simultaneously, the weak influence of cultural difference indicates that Wushu, as a form of “body language,” possesses comprehensibility that transcends cultural boundaries (14). Through visual media presentation, the aesthetics of Wushu movements themselves can effectively reach audiences from different cultural backgrounds (86), thereby reducing the necessity for cultural decoding.

Second, communicator characteristics also merit attention. The negative correlation effect exhibited by social capital contradicts common expectations—typically, creators with high follower counts are expected to receive more positive audience feedback (62). This “niche creator advantage” may reflect audience preference for authenticity: smaller creators are seen as genuine Wushu practitioners and cultural custodians, not commercially motivated influencers (61). In contrast, content supply demonstrates positive influence, supporting findings from Instagram opinion leader research (35). This is mainly because consistent content creation helps build audience trust, thereby enhancing their positive value perception.

Third, regarding narrative elements, culture image emerges as the second most important among all factors. Chinese Wushu artist images contribute to enhancing audience perceived value, reflecting Wushu short video audiences' preference for “authentic” Eastern cultural presentation. This phenomenon may arise because Wushu short videos featuring authentic Chinese Wushu artist images can satisfy audiences' expectations for cultural authenticity, thereby generating cultural curiosity psychology (72). Concerning character gender, female Wushu artists or mixed-gender displays contribute more to audience perceived value than male artists. This contradicts the negative evaluation phenomenon of female identity on YouTube (70). A possible explanation is that Wushu has traditionally been perceived as a male-dominated practice. Female participation disrupts this framework, highlighting its inclusivity and diversity, which in turn appeals to a wider audience. Theme type and theme depth characteristics have weak influence on audience perceived value, possibly due to audiences' unfamiliarity with Wushu culture (86), making it difficult for them to understand cultural differences across different thematic levels.

Finally, regarding multimodal features, visual richness exhibits certain positive influence, confirming the effectiveness of visual content (101). Audio loudness in Wushu short videos exhibits a U-shaped pattern, which contrasts with findings from studies on hotel influencers' content (41). This difference may stem from the unique nature of Wushu: low volume evokes a calm, distant artistic atmosphere, while high volume emphasizes strength and impact. Both can enhance the viewing experience in distinct ways. Overall, multimodal feature influences remain quite limited, possibly because existing Wushu short videos show minimal differences in audiovisual quality, making it difficult to influence audience value judgments.

### 5.2 Theoretical implications

This study makes several theoretical contributions. First, this research identified the multidimensional structure of audience perceived value in Wushu short videos. Addressing the gap in existing research that lacks exploration of Wushu video communication from the audience perspective, this study identified three dimensions through large-scale comment text mining: knowledge expansion value, physical health value, and emotional experience value. Building upon the validation of the two established dimensions of informational value and entertainment value from previous sports video research (20, 58), this study further revealed the continuation of health cultivation concepts from Wushu culture in the digital media environment. This finding confirms a unique attribute of Wushu and enriches the theoretical understanding of value perception in sports cultural content.

Second, this study uncovered the complex mechanisms underlying factors that influence audience perceived value in Wushu short videos. Drawing on the 5W communication model, we systematically examined the effects of communicator competence, narrative elements, multimodal features, and audience cultural background. The analysis revealed varying degrees of importance among different factors and identified several counterintuitive patterns. For example, cultural diversity demonstrated dominant effects compared to cultural difference, while communicator social capital exhibited negative impacts. These findings suggest the need to reconsider existing perspectives on “cultural difference impediment effects” and “social capital advantages” (62, 108). They reveal the complex mechanisms of value perception in Wushu short videos and provide a rich empirical foundation for future research on the digital communication of Wushu and related sports cultures.

Finally, this study offers methodological contributions to digital communication research in sports culture. By collecting video content and user comments from TikTok and applying BERT-based semantic analysis, LightGBM modeling, and SHAP interpretability techniques, we identified key dimensions of audience perceived value and unpacked the mechanisms through which various communication factors exert influence. This data-driven machine learning approach moves beyond the constraints of traditional surveys and interviews, demonstrating the viability of computational methods in studying sports cultural content on digital platforms and providing a replicable analytical framework for future research.

### 5.3 Practical implications

This study offers several practical implications based on the four key factors influencing audience perceived value. First, in terms of communicator competence, the positive effect of content supply suggests that maintaining a regular publishing schedule can enhance perceived value. In contrast, the negative correlation with social capital indicates that smaller creators may hold an “authenticity advantage” over influencers with large followings. These findings imply that creators should prioritize showcasing their genuine Wushu expertise rather than focusing on follower quantity, while also maintaining consistent content output to build credibility and trust.

Second, concerning narrative elements, the findings reveal that cultural image is the second most important factor, with Chinese Wushu artist imagery significantly enhancing audience perceived value compared to non-Chinese representations. Character gender also demonstrates substantial influence, where female or mixed-gender presentations outperform traditional male-dominated displays. Based on these, creators should emphasize authentic Chinese cultural elements through traditional costumes, classical settings, and cultural symbols to satisfy international audiences' expectations for genuineness. Additionally, creators should incorporate more female Wushu artists to break traditional stereotypes and attract broader audiences.

Third, regarding multimodal features, the findings indicate that visual richness positively influences perceived value, while audio elements have varied effects on audience engagement. Specifically, audio tempo shows a clear positive correlation with perceived value, whereas audio loudness exhibits a U-shaped relationship with audience response. Based on these results, creators should enhance visual appeal. For audio design, fast-paced background music can help stimulate positive emotional experiences. Furthermore, volume levels should be strategically adjusted to align with content type—using lower volumes in contemplative Tai Chi demonstrations to evoke a meditative atmosphere, and higher volumes in dynamic combat scenes to amplify intensity and viewer engagement.

Finally, concerning audience cultural background, the finding that cultural diversity dominates over cultural difference reveals that multicultural interaction environments enhance perceived value critically. Therefore, creators should focus on cultivating multicultural exchange atmospheres by actively encouraging diverse audience participation, responding to comments from different cultural perspectives, thereby fostering inclusive Wushu community engagement.

### 5.4 Conclusion

As Wushu culture continues to expand globally through digital platforms, understanding how audiences perceive and engage with Wushu short videos has become increasingly important. From the perspective of audience perceived value, this study draws upon Lasswell's 5W communication model to systematically investigate four key elements: communicator competence, narrative elements, multimodal features, and audience cultural background. We collected Wushu short videos and user comments from the TikTok platform to examine how viewers perceive the value of these videos and explore the impact of different communication factors on audience perception. Using topic clustering and sentiment analysis techniques, the study identified three dimensions of audience perceived value: knowledge expansion value, physical and health value, and emotional experience value, revealing differentiated emotional attitudes across these dimensions. Further, through interpretable machine learning, the key factors influencing audience perceived value and their mechanisms were analyzed. For instance, the study found that cultural diversity is a primary driver of enhancing audience perceived value, while Chinese Wushu artist imagery significantly strengthens the perceived value. In contrast, the communicator's social capital was found to have a negative impact. Meanwhile, elements such as theme depth, theme type, and subtitle type had relatively weak effects on audience value perception. Together, these findings advance theoretical understanding of digital Wushu cultural communication and offer content creators evidence-based guidance for optimizing dissemination strategies.

### 5.5 Limitations and future research

While this study provides valuable insights into audience perceived value of Wushu short videos, several limitations warrant acknowledgment that future research could address. First, machine learning techniques effectively identified main effects but have limitations in capturing potential interaction effects among variables. Future research could employ structural equation modeling to explore the interaction effects between different communication elements. Second, our study relied exclusively on TikTok data, which may limit generalizability across different social media environments. In addition, audience cultural background was inferred from comment language, which may not fully reflect users' native cultural identities on global platforms where English is widely used. Future research could extend investigations to other platforms such as YouTube Shorts and Instagram Reels to examine whether findings remain consistent across diverse digital ecosystems. Third, while involving international audiences, we did not conduct systematic cross-cultural comparative analysis, as the mechanisms of how Eastern and Western audiences differently perceive Wushu content remain underexplored. Future research could employ multi-group analysis to compare differential value perception mechanisms across cultural regions.

## Data Availability

The raw data supporting the conclusions of this article will be made available by the authors upon request. Requests to access the datasets should be directed to SX, feirulie717@163.com.
